# Effects of MAP4K inhibition on neurite outgrowth

**DOI:** 10.1186/s13041-023-01066-2

**Published:** 2023-11-18

**Authors:** Di Ja Lasham, Reza K. Arta, Abdul Fuad Hadi, Jun Egawa, Vance P. Lemmon, Toshiyuki Takasugi, Michihiro Igarashi, Toshiyuki Someya

**Affiliations:** 1 Departments of Psychiatry, School of Medicine, and Graduate School of Medical and Dental Sciences, Niigata University, 757 Asahimachi Dori-Ichibancho, Chuo-Ku, Niigata, 951-8510 Japan; 2 Departments of Neurochemistry and Molecular Cell Biology, School of Medicine, and Graduate School of Medical and Dental Sciences, Niigata University, 757 Asahimachi Dori-Ichibancho, Chuo-Ku, Niigata, 951-8510 Japan; 3 Miami Project to Cure Paralysis, University of Miami Miller School of Medicine, Miami, FL USA; 4 Institute for Data Science and Computing, University of Miami Miller School of Medicine, Miami, FL USA

**Keywords:** Mitogen-activated protein kinase kinase kinase kinases (MAP4Ks), High content screening, Neurite growth, Synaptogenesis, Psychiatric disorders

## Abstract

Protein kinases are responsible for protein phosphorylation and are involved in important intracellular signal transduction pathways in various cells, including neurons; however, a considerable number of poorly characterized kinases may be involved in neuronal development. Here, we considered mitogen-activated protein kinase kinase kinase kinases (MAP4Ks), related to as candidate regulators of neurite outgrowth and synaptogenesis, by examining the effects of a selective MAP4K inhibitor PF06260933. PF06260933 treatments of the cultured neurons reduced neurite lengths, not the number of synapses, and phosphorylation of GAP43 and JNK, relative to the control. These results suggest that MAP4Ks are physiologically involved in normal neuronal development and that the resultant impaired neurite outgrowth by diminished MAP4Ks’ activity, is related to psychiatric disorders.

**Supplementary Information:**

The online version contains supplementary material available at 10.1186/s13041-023-01066-2.

Neuronal outgrowth and synaptogenesis transpire during prenatal, the upstream processes of axonal circuitry complexes [1]. Yet, dysregulation of these could lead to microscopic changes, which are commonly found in psychiatric disorders [2]. Molecular cues such as protein kinases, play some important roles to guide axonal outgrowth and synapse formation during neurodevelopment, via protein phosphorylation in signal transduction [3–5].

In the course of neurogenesis, mitogen-activated protein kinase (MAPK) signaling is the common pathways of several molecules to facilitate proliferation of neural progenitors and their differentiation [6]. Some of these molecules belong to the mitogen-activated protein kinase kinase kinase kinases (MAP4K) family that act as the upstream signals for the MAPK cascade [7]. Among seven members of MAP4K family [8, 9], namely, MAP4K4/ NIK [10], MAP4K6/ MINK1, and MAP4K7/ TNIK are in close homolog, particularly 90% identical between MAP4K4 and TNIK in kinase domain and germinal center kinase homology domain, and 86.8% homolog between MAP4K4 and MINK1 [11, 12]. Despite the almost utterly identical structure, a line of evidence demonstrated TNIK is linked to schizophrenia, whereas MINK1 and MAP4K4 are associated with autism [13–18].

A downstream effect of the three MAP4K members regulate DLK-dependent JNK signaling and supports the translocation of activated JNK to nucleus of neuron [19]. Furthermore, phosphoproteomic analysis reveals that phosphorylated Ser96 GAP43 is abundant during brain development and strongly regulated by JNK-1 and JNK-2, indicating a novel molecular marker for developing axons [20]. Taken together, we hypothesize that MAP4Ks affects the following sequential phosphorylation cascades; from phosphorylated JNK to phosphorylated GAP43 in neurite and synapse formation (Fig. 1A).

**Fig. 1:**
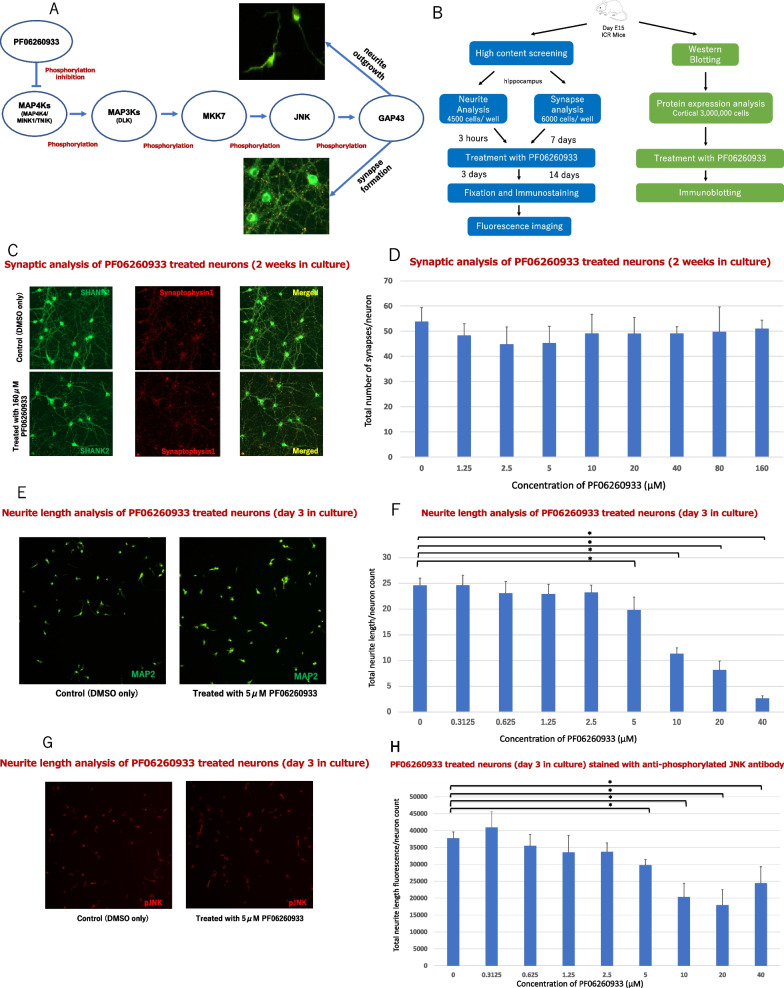

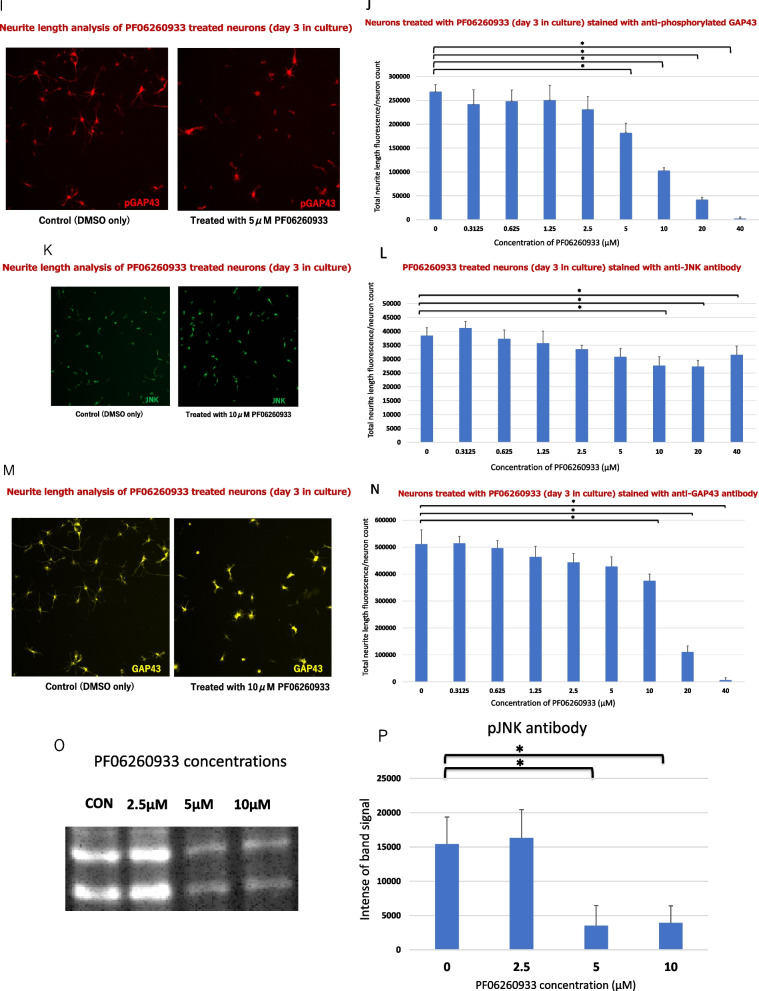

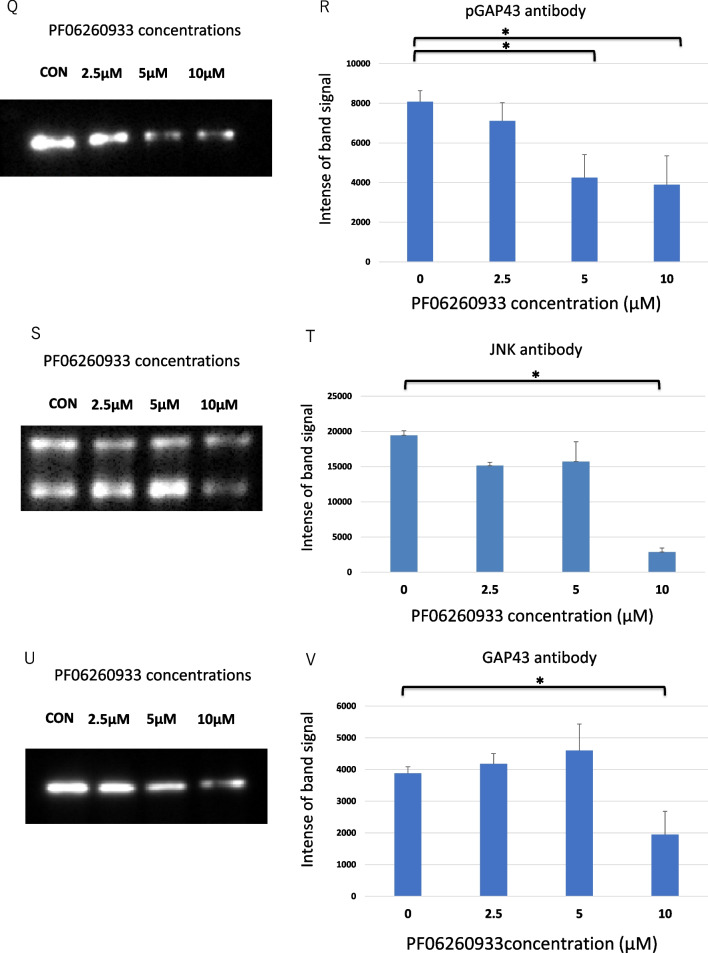
inhibition effects of MAP4Ks in the development of synapses and neurites. **A** The diagram of a hypothesized MAP4Ks-JNK-GAP43 cascade. According to ref. 20, brain-specific MAP2K for JNK is thought to be MKK7. **B** The workflow of the study methods. **C** Cell imaging: Synaptic analysis of neurons (2 weeks in culture) treated with DMSO (control) and with 160 μM PF06260933, immunostained by SHANK2 and synaptophysin. **D** Synaptic analysis of two-weeks old neurons treated with DMSO, and 1.25, 2.5, 5, 10, 20, 40, 80, and 160 μM PF06260933. **E** Cell imaging: Analysis of neurite lengths (day 3 in culture) treated with DMSO and with 160 μM PF06260933, immunostained by MAP2. **F** Neurite analysis of day 3 old MAP2-immunostained neurons treated with DMSO, and 0.3125, 0.625, 1.25, 2.5, 5, 10, 20, and 40 μM PF06260933 (eight different concentrations for neurite analysis). **G** Cell imaging: Neurite length analysis treated with DMSO or 5 μM PF06260933, immunostained using anti-phosphorylated JNK (pJNK) antibody. **H** Analysis of neurites on three-days old using pJNK-immunostained neurons, treated with DMSO or PF06260933 (the same concentrations as in **G**). **I** Cell imaging: Neurite length analysis treated with DMSO and 5 μM PF06260933, immunostained using phosphorylated GAP43 (pGAP43) antibody. **J** Neurite analysis of day 3 old pGAP43-immunostained neurons treated with DMSO and PF06260933 (same concentrations as in **G**). **K** Cell imaging: Neurite length analysis treated with DMSO and 10 μM PF06260933 enhanced by JNK immunostaining. **L** Neurite analysis of three-days old JNK-immunostained neurons treated with DMSO and PF06260933 (same concentrations as in **G**). **M** Cell imaging: Neurite length analysis treated with DMSO and 10 μM PF06260933 enhanced by GAP43 immunostaining. **N** Neurite analysis of day 3 old GAP43-immunostained neurons treated with DMSO and PF06260933 (same concentration as in **G**). **O**–**V** Protein expression in cells treated with control, and 2.5, 5, and 10 μM PF06260933 in gross protein visualization of pJNK (**O**), pGAP43 (**Q**), JNK (**S**), and GAP43 (**U**); and signal intensity quantification of pJNK (**P**), pGAP43 (**R**), JNK (**T**), and GAP43 (**V**). Significant comparison is marked with (*) at p < 0.05/8 (**D, F, H, J, L, N**; n = 6 each concentration) and at p < 0.05/3 (**P, R, T, V**; n = 3 each concentration), and error bars represent standard deviation

To investigate the MAP4Ks effect on neurite outgrowth and synapse formation, we used PF06260933 as a MAP4Ks inhibitor common to MAP4K4 (IC50 = 140 nM), MINK1 (IC50 = 8 nM), and TNIK (IC_50_ = 15 nM) to treat the cultured mouse hippocampal neuron [21, 22]. The simplified experiment workflow is depicted in Fig. 1B (the details are described in our previous work with the exception of PF0626093 treatments, staining and western blot protocols are described in Additional file 1) [23]. A high-throughput screening system, the microscope-based CellInsight™ CX5 High Content Screening platform (Thermo Fisher Scientific), was utilized to acquire the quantification data.

First, we compared the total number of synapses per a total number of neurons given eight different concentrations of PF06260933 compared to the control by using immunostaining of synaptophysin (presynaptic marker), SHANK2 (postsynaptic marker), and MAP2 (neurite marker). In our observations, no significant discrepancy across all concentrations compared to the control treated with DMSO (*p* > 0.05/8; Fig. 1C, D). In stark contrast, the total neurite length per neuron measured by MAP2 immunostaining, was dramatically reduced in 5 μM or more PF06260933, compared to the DMSO-supplemented control (*p* < 0.05/8; Fig. 1E, F). The results of the neurite analysis brought our investigation further into the JNK conditions in neurons.

Next, the PF06260933 effects on JNK and GAP43 signals (as JNK downstream effects) were scrutinized by the observation of the intensity of JNK/GAP43, and the ratios of (phosphorylated JNK1 and JNK2)/ (phosphorylated GAP43) per neuron were calculated in eight different PF06260933 concentrations, compared to the control. It was apparent that a significant reduction of neurite-derived fluorescence was observed in both phosphorylated JNK and phosphorylated GAP43 at 5 μM or more PF06260933-treated neurons, compared to control (*p* < 0.05/8; Fig. 1G–J). Similarly, the neurite-derived fluorescence of JNK/ GAP43 signals also dropped significantly at 10 μM or more, compared to the control (*p* < 0.05/8; Fig. 1K–N).

To validate these imaging results, we performed western blotting to evaluate the alteration of JNK/ GAP43 and (phosphorylated JNK1 and JNK2 protein)/ pS96 GAP43. Phosphorylated JNK1 and JNK2/ pS96 GAP43 demonstrated a significant decline of protein abundance at 5 μM and 10 μM both quantitively (*p* < 0.05/3; Fig. 1O–R). JNK/ GAP43 also resulted a significant decline of protein abundance at 10 μM both quantitively (p < 0.05/3; Fig. 1S–V). We have shown the original membrane photographs and the quantitative data for each band of the Western blotting experiments (Additional file 1: Fig. S1 and Table S1).

Based on our hypothesis, we showed in this study that the MAP4Ks were involved in neurodevelopment through the MAP4Ks-JNK-GAP43 cascade by observing the effects of the MAPKs inhibitor PF06260933. We also found that the MAP4Ks facilitate the neurite outgrowth but not synaptogenesis (Fig. 1C, D). Our current results were consistent with a previous report of MAP4K7/ TNIK knockout mice, which have a major reduction of dentate gyrus neurons in number, due to affect the cytoskeletons [24]. On the other side, synapse formation requires synaptic organizing molecules that orchestrate the synaptic establishment and the synapse shapes, by which various signals are craved [25].

In addition, our findings support that the hypothesized cascade of the MAP4Ks accommodates neurite outgrowth via JNK-GAP43 phosphorylation fashion. JNK and GAP43 signals had similar results (Fig. 1L–O). Likewise, pJNK and Ser96 pGAP43 demonstrated the exact concentration at which the intensity began to be affected (Fig. 1G–J). In other words, both the neurite intensity of the pJNK and that of the pGAP43 are similar to each other after the administration of MAP4Ks inhibitor, indicating the strong association between the two in neurite development as also previously reported [20, 26].

As mentioned above, PF06260933 inhibits the similarly structured MAP4K members. First, MAP4K4 has a number of tissue-specific isoforms and is one of the autism spectrum disorders (ASD) genes after identifying de novo frameshift variants in neuron-specific exons and isoforms, which account for the reduced expression of MAP4K4 transcript [17]. Similarly, a study reported MINK1 haploinsufficiency is the causal agent of the autistic manifestations in an ASD patient [18]. Also, TNIK-deficit mice demonstrated cognitive dysfunction [24], which is one of the signs of schizophrenia.

To note, inhibiting MAP4Ks, not individually, hinders the activation of JNK signaling mediated by stress-inducing conditions, and it provides neuroprotection from degeneration [19]. Although variants of the GAP43 gene are not associated with any neurodevelopmental disorders [27], deficit phosphorylated GAP43 in neurons greatly contributes to memory impairments, as previously reported [28, 29]. In brief, our study suggests the fact that an inhibitor of MAP4Ks obliterates neurite growth associated with the suppression of phosphorylated JNK-GAP43, implying the existence of a MAP4Ks-JNK-GAP43 cascade, which could lead to cognitive impairments of neurodevelopmental disorders. Further work is needed to establish the cascade distinctively and to detect other possible protein interactions that result in our association.

### Supplementary Information

**Additional file 1**. **Methods**, **Fig. S1** and **Table S1.**

## Data Availability

All data generated or analyzed during this study are included in this published article.
