# Supplementary material for: Effects of MAP4K inhibition on neurite outgrowth
Source: Mol Brain. 2023 Nov 18;16:79. doi: 10.1186/s13041-023-01066-2 (PMC10656890; doi:10.1186/s13041-023-01066-2)
Supplement: Supplementary file 1 — Additional file 1. Methods, Fig. S1 and Table S1. [file 13041_2023_1066_MOESM1_ESM.docx]

**Supplemental Information**

**Supplemental Methods**

***Treatment with PF06260933 as MAP4Ks Inhibitor***

PF06260933 were purchased from Bio-Techne/TOCRIS Japan. Plates were incubated for 3 hours in 95% humidity at 37°C prior to the addition of PF06260933 (in 0.8% DMSO) to each well. Treatments were given in accordance with previous protocol (20) with the adjustment of PF06260933 final concentrations of 0.3125, 0.625, 1.25, 2.5, 5.0, 10, 20, and 40 µM for neurite analysis, and of 1.25, 2.5, 5.0, 10, 20, 40, 80, and 160 µM for synapse analysis; 0.8% DMSO was added to the control wells.

***Staining***

Cultured neurons were fixed and blocked prior to staining following our previous work (20). Primary antibody staining was performed overnight at 4°C in the same buffer as elaborated in Egawa et al 2022, containing antibodies that recognized MAP2 (1:300, Cat# PA5-85755, rabbit, Invitrogen) only for neurite length analysis, or synaptophysin 1 (1:400, Cat# 101 011, mouse, Synaptic Systems), SHANK2 (1:400, Cat# 162204, guinea pig, Synaptic Systems), and MAP2 (1:300, Cat# PA5-85755, rabbit, Invitrogen) for synapse number analysis. In addition, anti-GAP43 (1:500, Cat#8945, rabbit, Cell Signaling Technology), anti-p-GAP43(S96) (1:300, clone 18-10H-9H, mouse, FUJIFILM Wako), anti-JNK (1:200, sc-7345, mouse, Santa Cruz Biotechnology), and anti-pJNK1+pJNK2(T183+Y185) (1:1000, ab4821, rabbit, Abcam) were used to investigate the hypothesized MAP4Ks-JNK-GAP43 cascade. The next day, the cells were washed three times with PBS, incubated for 2 hours at room temperature with fluorescently labeled secondary antibodies (1:500, Cat# 706-545-148, donkey anti-guinea pig-IgG (H+L)-Alexa488, Jackson IR; 1:500, Cat# A10042, donkey anti-rabbit-IgG (H+L)-Alexa568, Invitrogen; 1:500, Cat# A31571, donkey anti-mouse-IgG (H+L)-Alexa647, Invitrogen) and 1:1000 DAPI. The cells were then washed five times with PBS. All washing steps were performed with a robotically operated Wellwash Versa (Thermo Fisher Scientific). Brightfield, nuclei, and GFP images of the cells were then acquired with the microscope-based CellInsight^TM^ CX5 High Content Screening (HCS) platform (Thermo Fisher Scientific) using a 10X (for neurite length analysis) or 20X (for synapse number analysis) objective lens.

**Western Blot**

We deployed a western blot technique to cultivate three million cortical neuron culture treated with three distinct MAP4Ks inhibitor concentrations (2.5 μM, 5 μM, and 10 μM) to visually assess the relative abundance of JNK/ GAP43 and the phosphorylated JNK/ GAP43 proteins against the cell culture treated with DMSO alone. To denature proteins and yield them negatively charged, we stored the collected cells at -20 °C using 2X SDS sample buffer. Then, 2-Mercaptoethanol was added to a final 5% to break protein bonds. Next, we heated the cells at 95 °C for 5 minutes. Following this, we stored the cells at 4 °C. Electrophoresis was run with cc.10 mA/ gel to move proteins to the bottom of stacking gel immersed with SDS running buffer. The current was consequently increased to cc.20 mA/ gel for about 90 minutes. Lastly, we transferred the proteins from the gel to a PVDF membrane, which was previously prepared in methanol for 1-2 minutes and in the transfer buffer sequentially, and we stacked two sheets of filter papers onto one side of the PVDF membrane and two sheets of filter papers onto one side of the gel, with the current cc.100mA.

The PVDF membrane then was soaked in anti-GAP43 1:1000 (Cell Signaling Technology), anti-p-GAP43(S96) 1:1000 (FUJIFILM Wako), anti-JNK 1:1000 (Santa Cruz Biotechnology), and anti-pJNK1+pJNK2(T183+Y185) 1:1000 (Abcam) as primary antibodies, and Alexa 488 1:500 (Jackson IR), Alexa 568 1:500 (Invitrogen), and Alexa 647 1:500 (Invitrogen) as secondary antibodies for protein visualization.

To analyze the expression level of GAP43, JNK, pGAP43, and pJNK, ImageJ software Fiji (http://rsb.info.nih.gov/ij) was used to measure the areas under the curve of the immunoblotted bands.

**Supplemental data**

Figure S1, The original membrane pictures of western blotting experiments

1. Anti pJNK antibody


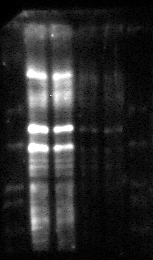


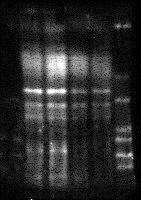


Experiment II

Experiment I


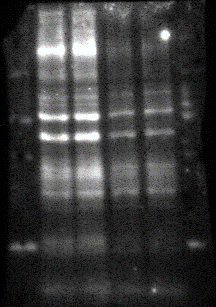


Experiment III

1. Anti pGAP43 antibody


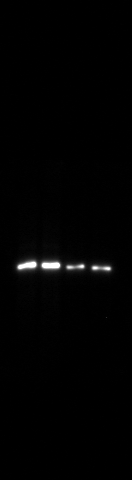


Experiment III

Experiment II


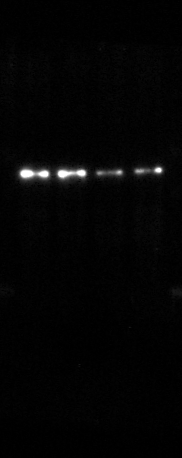


Experiment I


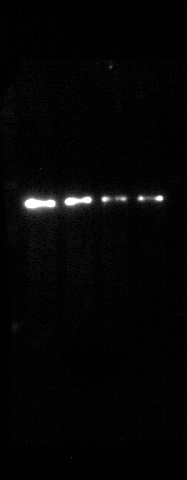


1. Anti JNK antibody


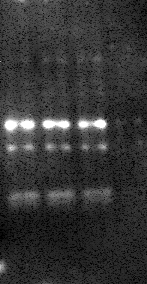


Experiment III

Experiment I


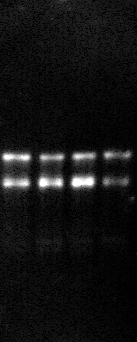


Experiment II


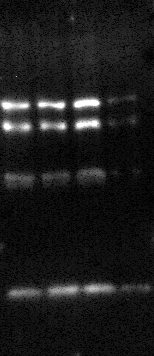


1. Anti GAP43 antibody

Experiment II

Experiment II

Experiment I


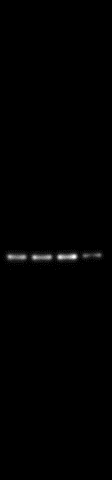

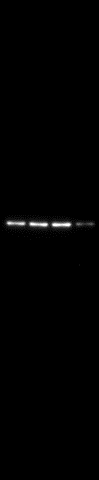

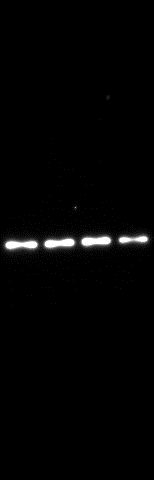


**Table S1, Quantitative data for each band in Western blotting experiments.**

1. Anti pJNK antibody

| PF06260933 concentrations (μM) | 0 | 2.5 | 5 | 10 |
| --- | --- | --- | --- | --- |
| Intense of band signal (Expt. I) | 19461.14 | 20633.77 | 6730.48 | 6643.439 |
| Intense of band signal (Expt. II) | 11546.95 | 15947.42 | 2909.468 | 3397.711 |
| Intense of band signal (Expt. III) | 15208 | 12414.24 | 918.184 | 1743.539 |
| Mean | 15405.36 | 16331.81 | 3519.377 | 3928.23 |
| SD | 3960.782 | 4123.228 | 2953.758 | 2492.658 |

1. Anti pGAP43 antibody

| PF06260933 concentrations (μM) | 0 | 2.5 | 5 | 10 |
| --- | --- | --- | --- | --- |
| Intense of band signal (Expt. I) | 8459.709 | 6062.848 | 3392.927 | 2350.56 |
| Intense of band signal (Expt. II) | 8334.421 | 7478.522 | 5576.43 | 5220.309 |
| Intense of band signal (Expt. III) | 7434.912 | 7788.912 | 3773.962 | 4118.426 |
| Mean | 8076.347 | 7110.094 | 4247.773 | 3896.432 |
| SD | 559.0203 | 920.1242 | 1166.316 | 1447.697 |

1. Anti JNK antibody

| PF06260933 concentrations (μM) | 0 | 2.5 | 5 | 10 |
| --- | --- | --- | --- | --- |
| Intense of band signal (Expt. I) | 18671.55 | 15094.84 | 15472.14 | 3058.205 |
| Intense of band signal (Expt. II) | 19965.92 | 15637.97 | 18628.67 | 3283.439 |
| Intense of band signal (Expt. III) | 19638.19 | 14620.84 | 13033.19 | 2181.489 |
| Mean | 19425.22 | 15117.88 | 15711.33 | 2841.044 |
| SD | 672.9498 | 508.9523 | 2805.401 | 582.1877 |

1. Anti GAP43 antibody

| PF06260933 concentrations (μM) | 0 | 2.5 | 5 | 10 |
| --- | --- | --- | --- | --- |
| Intense of band signal (Expt. I) | 3902.648 | 3803.113 | 3737.406 | 2647.163 |
| Intense of band signal (Expt. II) | 3670.376 | 4433.619 | 4658.619 | 1175.82 |
| Intense of band signal (Expt. III) | 4075.062 | 4283.77 | 5406.062 | 2009.234 |
| Mean | 3882.695 | 4173.501 | 4600.696 | 1944.072 |
| SD | 203.0795 | 329.3994 | 835.8346 | 737.8327 |
